# Effects of the Mg Content on Microstructural and Corrosion Characteristics of Hot-Dip Al–Si–Mg Alloy-Coated Steel Sheets

**DOI:** 10.3390/ma16175827

**Published:** 2023-08-25

**Authors:** Kwang-Hyeok Jin, Min-Suk Oh

**Affiliations:** Division of Advanced Materials Engineering, Research Center of Advanced Material Development, Jeonbuk National University, Jeonju 54896, Republic of Korea; jindory5435@hanmail.net

**Keywords:** hot-dip coating, Al–Si coated steel, Al_3_Mg_2_, corrosion resistance

## Abstract

Hot-dip Al–Si alloy coatings with excellent resistance to corrosion and high-temperature oxidation have emerged as promising lightweight substitutes for conventional corrosion-resistant coatings. The introduction of Mg can be an effective strategy for enhancing the sacrificial protection capability of Al–Si coatings. In this study, the effects of Mg addition on the morphology, electrochemical behavior, and mechanical properties of Al–Si coatings were investigated, along with the Mg-content optimization of the coating layer. Adding Mg promoted the formation of finely distributed eutectic intermetallic phases, such as Al/Mg_2_Si and the primary Mg_2_Si phase. Notably, the Mg_2_Si phase coarsened significantly when ≥15 wt.% of Mg was added. In addition, an Al_3_Mg_2_ intermetallic compound was observed in coating layers containing >20 wt.% of Mg, reducing the adhesion of the coating layers. Samples containing 5–10 wt.% of Mg exhibited excellent corrosion resistance (owing to a uniform distribution of the fine eutectic Al/Mg_2_Si phase and the formation of stable corrosion products), whereas those containing 20 wt.% of Mg exhibited unremarkable corrosion resistance (owing to the formation of an Al_3_Mg_2_ phase that is susceptible to intergranular corrosion).

## 1. Introduction

Hot-dip Al-coated steel sheets with excellent corrosion resistance, high-temperature durability, and thermal insulation properties are employed in several industrial applications, such as power plants, exhaust systems, and heating equipment [1,2,3]. Al-based coatings can be classified into two categories based on their composition: type-1 and type-2 [1,4]; type-1 coatings comprise Al–Si alloys with 7–11 wt.% of Si, while type-2 coatings comprise pure Al (with no Si) [4]. Although such coatings exhibit high corrosion resistance under atmospheric conditions owing to the formation of stable Al_2_O_3_ surface films, they are unstable in chloride-containing environments [1,5,6]. Moreover, type-2 Al coatings frequently exhibit delamination owing to the excessive growth of the brittle Fe–Al intermetallic-compound layer during processing [5]. Si addition improves the processability of type-1 Al–Si coatings by reducing the intermetallic-layer thickness by suppressing Fe–Al inter-diffusion [1]. Therefore, Al-Si coatings have been recently used as pre-coatings to prevent the severe oxidation and decarburization of steel during the hot-stamping process [7,8,9,10,11]. However, the limited solubility of Si in the Al matrix results in Si precipitation within these coating layers [12]. Moreover, the corrosion potential of Si (−0.17 V_SCE_ [13]) is higher than that of Al (−0.7 V_SCE_ [14]); the negative activity of Si reduces the corrosion resistance of the coating layer through localized corrosion [15,16].

Based on recent studies, the corrosion resistance of Al-based coatings can be effectively enhanced by including alloying elements, such as Mg and Zn, that modify the coating potential [5,6,7,17,18,19]. Kim et al. [4] examined the anti-corrosion mechanism of Al–Si coatings with small amounts of Mg (approximately 0.1–0.5 wt.%) and observed that the addition of Mg to Al–Si coatings increased their corrosion resistance in hot-press-forming applications [19]. Furthermore, the addition of 24 wt.% Zn to Al–10%Si coatings improved the corrosion resistance of the heat-treated Al–Si coatings [7]; this is because the introduction of Zn induces an additional self-healing effect by facilitating the sacrificial dissolution of the alloying elements [1,20,21]. Similarly, studies on Al–Si–Mg cast alloys have indicated that the addition of Mg into Al–Si alloys increases their corrosion resistance, owing to the formation of various intermetallic compounds [22,23,24]. The Mg_2_Si phase exhibits sacrificial-protection properties because of its negative corrosion potential (−1.54 V_SCE_) [5,17,25,26], and induces the formation of stable and dense Mg-based corrosion products, such as MgAl_2_O_4_, enhancing the corrosion-protection performance of Mg-added Al–Si cast alloys [27,28]. However, the addition of >20 wt.% of Mg into Al–4%Si alloys induces the formation of an Al_3_Mg_2_ phase [29], which is vulnerable to intergranular corrosion and stress-corrosion cracking [30,31,32,33,34], thereby reducing the corrosion resistance of the cast alloys. The aforementioned studies confirmed that the presence of Mg significantly influences the corrosion resistance of Al–Si alloys. Therefore, Mg content of Al–Si alloys must be optimized to facilitate their widespread application. To date, the correlation between the microstructural changes and corrosion resistance of hot-dip Al–Si-based coating layers with different Mg compositions has not been comprehensively analyzed. Consequently, to elucidate the influence of the Mg content of Al–Si-based alloy coatings on their properties, this study investigates the morphological properties, electrochemical-corrosion performance, and mechanical properties of hot-dip Al–Si–Mg ternary-alloy coating layers containing different amounts of Mg.

## 2. Materials and Methods

A 0.8 mm-thick commercial quality low-carbon cold-rolled steel sheet (POSCO Co., Ltd., Pohang, Republic of Korea) was used as the substrate. The nominal chemical composition (in wt.%) of the substrate was as follows: C (0.14), Mn (0.33), S (0.01), P (0.012), Si (0.05), Al (0.03), N (0.004), and Fe (Bal.). Each sample was cut into a specific size (150 × 30 mm^2^) and immersed in an Al–Si–xMg alloy coating bath of the required composition using a laboratory scale batch-type hot-dip simulator [35]. The alloy coating compositions were analyzed using an X-ray fluorescence spectrometer (listed in Table 1 with sample labels). Before hot-dipping, the substrates were degreased in NaOH solution (100 g/L) at 60 °C for 10 min to remove the impurities and residual oils on their surfaces. The samples were then rinsed with deionized water and pickled in 10% HCl at room temperature for 10 min to remove any retained oxides. These samples were then fluxed using ZnCl_2_·3NH_4_Cl·SnCl_2_ (550 g/L) at 60 °C for 3 min, followed by oven drying at 100 °C for 5 min to promote the reaction between the molten metal and the steel substrate [36]. All the samples were fabricated under identical coating conditions (a bath temperature of 730 °C and an immersion time of 1 min). The surface roughness was measured using a high-speed, three-dimensional (3D) laser confocal microscope (SURFiEW-PRO, GLtech, Daejeon, KOREA). Field-emission scanning electron microscopy (FE-SEM, Hitachi SU6600, Tokyo, Japan) coupled with energy dispersive spectrometry (EDS) was used to analyze the coating-layer surfaces and cross-sectional microstructures. A spherical aberration-corrected field-emission transmission electron microscopy (FE-TEM) instrument (JEOL-ARM200F) was used to investigate the elemental composition of the coatings. The coating-layer alloy phases were confirmed via X-ray diffraction (XRD, Rigaku RINT-2000, Tokyo, Japan) using monochromatic Cu-K_α_ radiation (λ = 0.15406 nm). The coating adhesion was evaluated through bending tests (according to the ASTM E290 standard [37]); the area fraction was estimated from the percentage of coating adhering to the tape after removal from the bent surface. The corrosion performance of each sample was analyzed using a potentiostat (GAMRY INTERFACE 1010E). A three-electrode system was employed with a saturated calomel electrode (SCE) as the reference electrode, graphite as the counter electrode, and the coated sample as the working electrode. Before electrochemical impedance spectroscopy (EIS) analysis, each sample was immersed in a NaCl solution (3.5 wt.%) for 1 h to ensure a stable open circuit potential (OCP). EIS was conducted from 100 kHz to 100 MHz (10 points per decade) with a 10 mV sinusoidal voltage perturbation and potentiodynamic polarization in the range of −0.3 to 0.5 V (vs. OCP), at a scan rate of 0.5 mV/s. The corrosion current density (i_corr_), corrosion potential (E_corr_), and corrosion rate (mpy) were evaluated via Tafel extrapolation; a built-in Gamry Echem Analyst software 7.10.0 module was used for the EIS Nyquist-plot curve fitting and analysis. Salt spray tests (SSTs, SUGA) were conducted at 35 °C (according to the ASTM B117 standard) to investigate the long-term corrosion behavior of the coatings. A constant-concentration NaCl spray (5 wt.%) at a flow rate of 1.5 ± 0.5 mL/h was used for the SSTs.

## 3. Results

### 3.1. Visual Inspection and Surface Roughness

**Figure 1:**
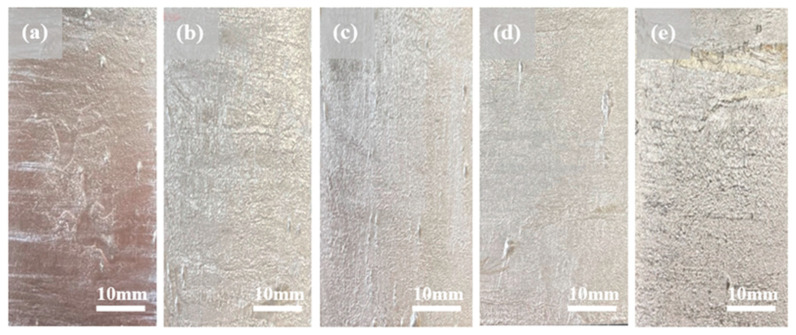
Photographs of steel sheets coated with the following Al–Si–xMg alloys: (**a**) AlSi; (**b**) AlSiMg5; (**c**) AlSiMg10; (**d**) AlSiMg15; (**e**) AlSiMg20.

**Figure 2:**
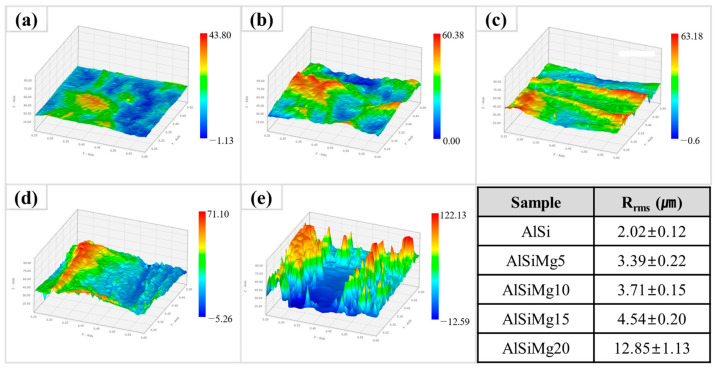
Three-dimensional (3D) surface topography of: (**a**) AlSi; (**b**) AlSiMg5; (**c**) AlSiMg10; (**d**) AlSiMg15; (**e**) AlSiMg20.

Photographs of the coated sample surfaces are shown in Figure 1. As shown in Figure 2, the addition of Mg increased the roughness of the Al–Si–xMg layer, reducing its surface glossiness; this phenomenon was quantitatively evaluated using 3D surface topography analysis, which was conducted on five distinct samples and the results were averaged. Al–Si coating layers with ≤15 wt.% and 20 wt.% of Mg showed slightly and significantly higher surface roughness, respectively, than the corresponding sample without Mg (AlSi). This can be primarily attributed to two factors: the formation of a thick Mg-oxide layer on the coating-bath surface and a reduction in the coating-bath fluidity [38].

### 3.2. Cross-Sectional Microstructure and Compositional Study

**Figure 3:**
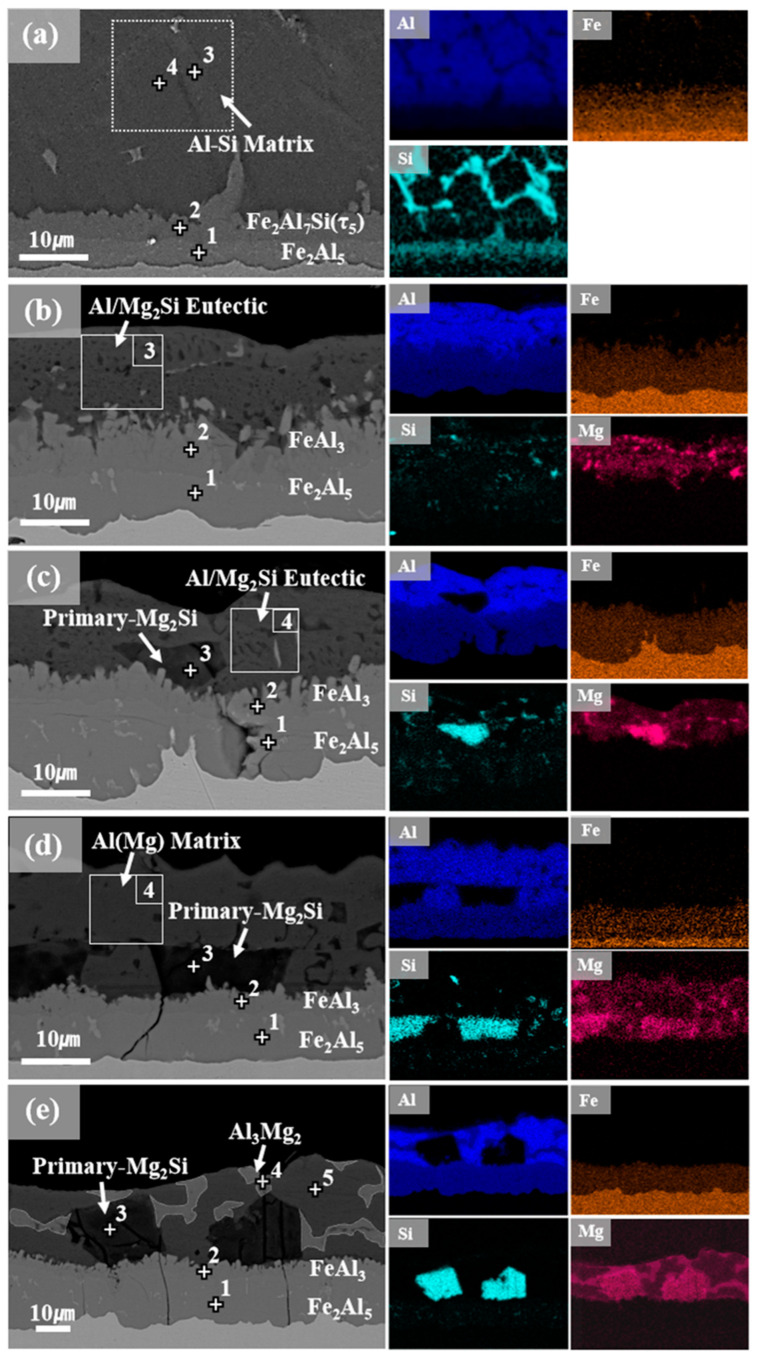
Cross-sectional microstructures with marked EDS points(+) of the following samples: (**a**) AlSi; (**b**) AlSiMg5; (**c**) AlSiMg10; (**d**) AlSiMg15; (**e**) AlSiMg20.

Cross-sectional microstructures of the Al–Si–xMg alloy coatings are shown in Figure 3; EDS analysis results of the points marked in Figure 3 are listed in Table 2. The average coating thicknesses were 44.1, 28.2, 27.4, 30.8, and 39.3 μm for AlSi, AlSiMg5, AlSiMg10, AlSiMg15, and AlMg20, respectively. The coating cross-sections of all the samples showed the presence of an interfacial intermetallic layer and an Al-rich top layer. As shown in Figure 3a, AlSi contained a top layer (comprising α-Al and Si) and two interfacial intermetallic layers (comprising Fe_2_Al_5_ and τ_5_, respectively). In contrast, the Mg-added samples contained an interfacial intermetallic layer comprising FeAl_3_ and Fe_2_Al_5_ phases. The transformation of τ_5_ into FeAl_3_ in the Mg-added samples can be attributed to Si consumption owing to the formation of the Mg_2_Si phase. The presence of a τ_5_ phase at the interface reduced the intermetallic-layer thickness of the alloy coatings [4]. Therefore, the Mg-added samples (without the τ_5_ phase) contained thicker intermetallic interfacial layers than AlSi. Additionally, the formation of a fine Al/Mg_2_Si eutectic mixture in the AlSiMg5 top layer was observed in Figure 3b. This eutectic-mixture phase was also observed to coexist with the coarse primary Mg_2_Si phase in AlSiMg10, as shown in Figure 3c. Moreover, Figure 3d,e show the coarse primary Mg_2_Si phase inside Al–Mg matrices in AlSiMg15 and AlSiMg20, respectively; both these samples contained high Mg contents (≥15 wt.%). Notably, EDS point and mapping analyses confirmed the formation of an additional Al–Mg intermetallic compound (IMC), Al_3_Mg_2_, in AlSiMg20 (Figure 3e). 

**Figure 4:**
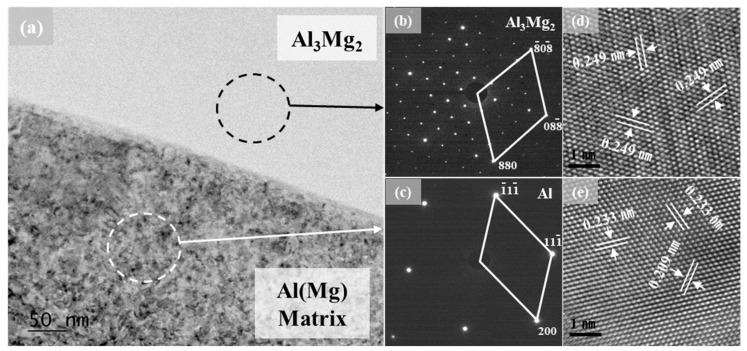
FE-TEM analysis of AlSiMg20: (**a**) TEM bright-field image; SAED patterns of the (**b**) Al_3_Mg_2_ phase and (**c**) Al phase; lattice images of the (**d**) Al_3_Mg_2_ phase and (**e**) Al phase.

For a comprehensive analysis of the Al–Mg IMC within AlSiMg20, the interface between the Al–Mg IMC and Al(Mg) matrix was analyzed using FE-TEM. Figure 4a shows the bright-field image of the interface. The selected area electron diffraction (SAED) patterns in Figure 4b,c indicate that the Al–Mg IMC has an Al_3_Mg_2_ phase and FCC-structured Al phase, respectively [39,40]. As shown in Figure 4d, the interplanar distance of the Al_3_Mg_2_ phase was 0.249 nm, corresponding to the (880) plane in all directions. Furthermore, the lattice image in Figure 4e indicates Al-phase interplanar distances of 0.233 and 0.209 nm, corresponding to the (111) and (200) planes, respectively.

### 3.3. Crystallographic Study of Alloy Coatings

**Figure 5:**
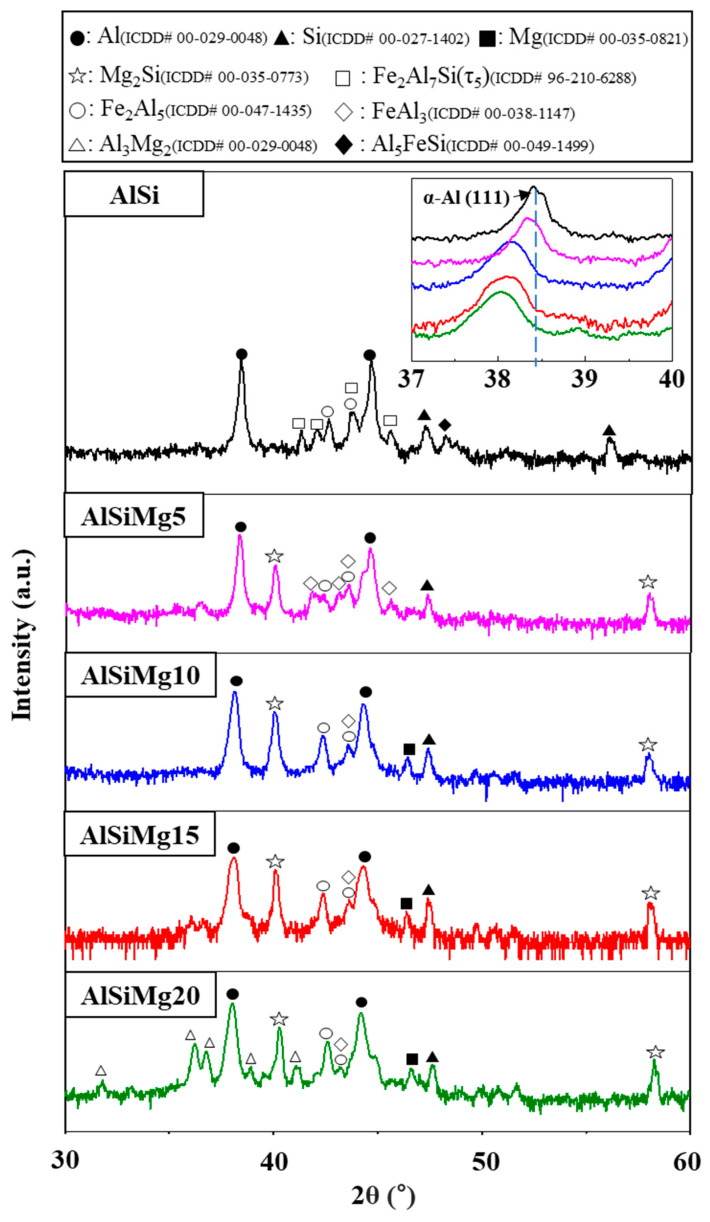
XRD patterns of Al–Si–xMg alloy coatings. The inset shows the magnified pattern of (111) plane.

XRD was used to investigate the effects of the Mg content on the crystallographic properties of the Al–Si–xMg alloy coatings; the corresponding patterns are provided in Figure 5. All the samples predominantly contained the α-Al phase, with additional peaks corresponding to the Mg_2_Si phase observed in the patterns of all the Mg-added samples. Our findings revealed a noticeable shift in the peak associated with the (111) crystallographic plane of α-Al toward lower scattering angles. This shift indicates the presence of a solid solution of Mg atoms within the Al-matrix, fostering lattice expansion [41,42]. Peaks corresponding to the Fe_2_Al_7_Si (τ_5_), Al_5_FeSi, and Fe_2_Al_5_ alloy phases were observed in the XRD pattern of AlSi, whereas the XRD patterns of the Mg-added samples indicated Mg_2_Si formation and the transformation of the τ_5_ phase into the FeAl_3_ phase. Furthermore, the addition of Mg resulted in the disappearance of the Fe_2_Al_7_Si (τ_5_) and Al_5_FeSi phases. Notably, the AlSiMg20 pattern contained peaks corresponding to the Al_3_Mg_2_ phases, which is consistent with the SEM–EDS results (Figure 3 and Table 2).

### 3.4. Study of Surface Microstructures and Composition

**Figure 6:**
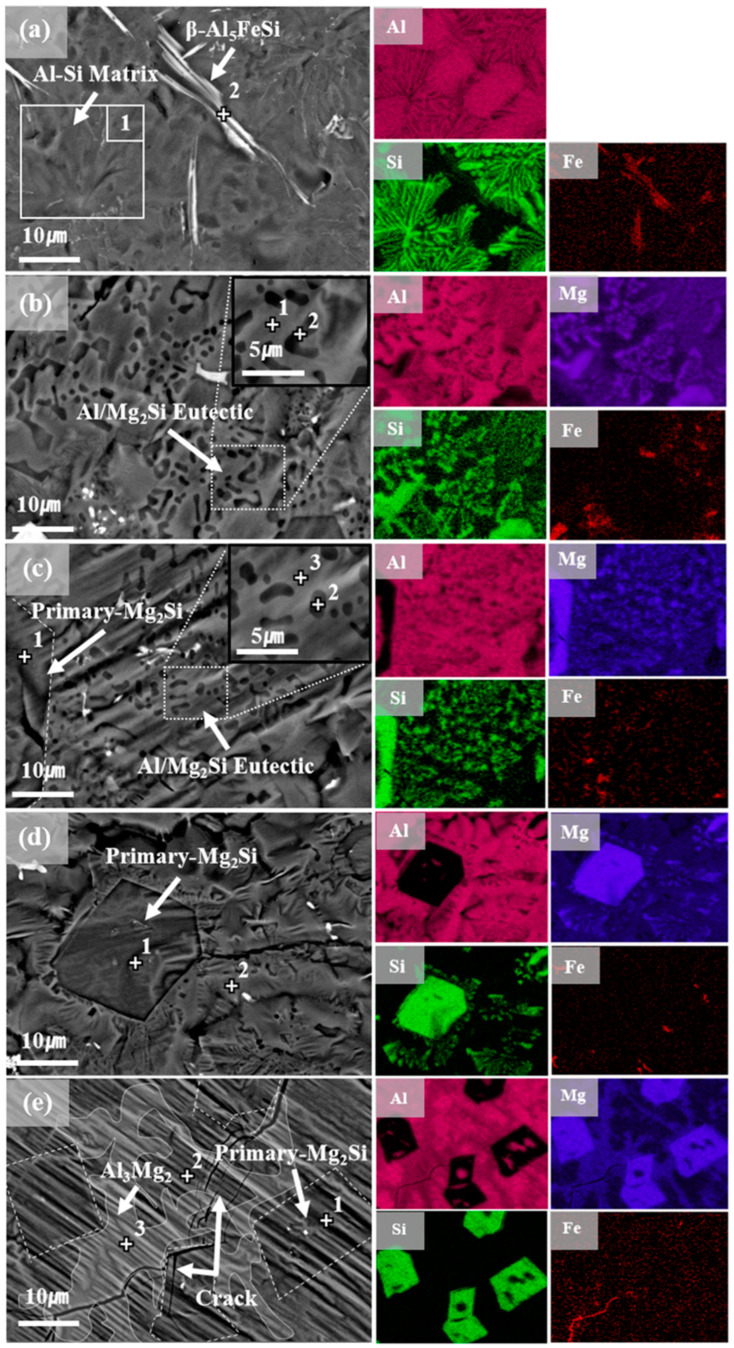
Surface microstructures with marked EDS points(+) and EDS phase maps of: (**a**) AlSi; (**b**) AlSiMg5; (**c**) AlSiMg10; (**d**) AlSiMg15; (**e**) AlSiMg20.

The surface microstructure affects the corrosion and mechanical performance of surface coatings; therefore, its analysis is crucial. FE-SEM images of the surface microstructures of all the samples are provided in Figure 6, and the results of the EDS analyses are listed in Table 3. A distinct formation comprising the acicular β-Al_5_FeSi phase was observed on the AlSi surface; this structure was embedded within an Al–Si eutectic lamellar matrix, in which Si is distributed in the interdendritic spaces of Al, as indicated by the EDS phase map shown in Figure 6a. Upon Mg addition, this coarse Al–Si eutectic lamellar structure transformed into a fine Al/Mg_2_Si eutectic mixture, as observed in the FE-SEM image of AlSiMg5. Figure 6c indicates a significant volume fraction of the coarse Mg_2_Si phase in the samples containing ≥10 wt.% of Mg. Moreover, a phase-map analysis of the Mg and Si EDS results confirms the coexistence of the fine Al/Mg_2_Si and coarse Mg_2_Si phases. As the amount of Mg in the samples increased (>15 wt.%), the volume fraction of the fine Al/Mg_2_Si eutectic-mixture phase gradually decreased, whereas the coarse primary Mg_2_Si phase emerged as the predominant constituent (Figure 6d) [43]. As shown in Figure 6e, the coarse primary Mg_2_Si phase completely replaced the Al/Mg_2_Si eutectic mixture in AlSiMg20. The EDS point analysis (points 1–3) and phase maps (Al and Mg) confirmed the formation of the Al_3_Mg_2_ phase. The Al_3_Mg_2_ phase, which forms along the grain boundaries, is inherently brittle and prone to cracking. Several surface cracks (extending up to the Fe substrate) were detected in AlSiMg20. Furthermore, discernible wrinkle-defect patterns were observed on the surface of AlSiMg20. The high roughness of the sample can be attributed to these wrinkle-defect patterns, based on the 3D surface topographical analysis shown in Figure 2.

**Figure 7:**
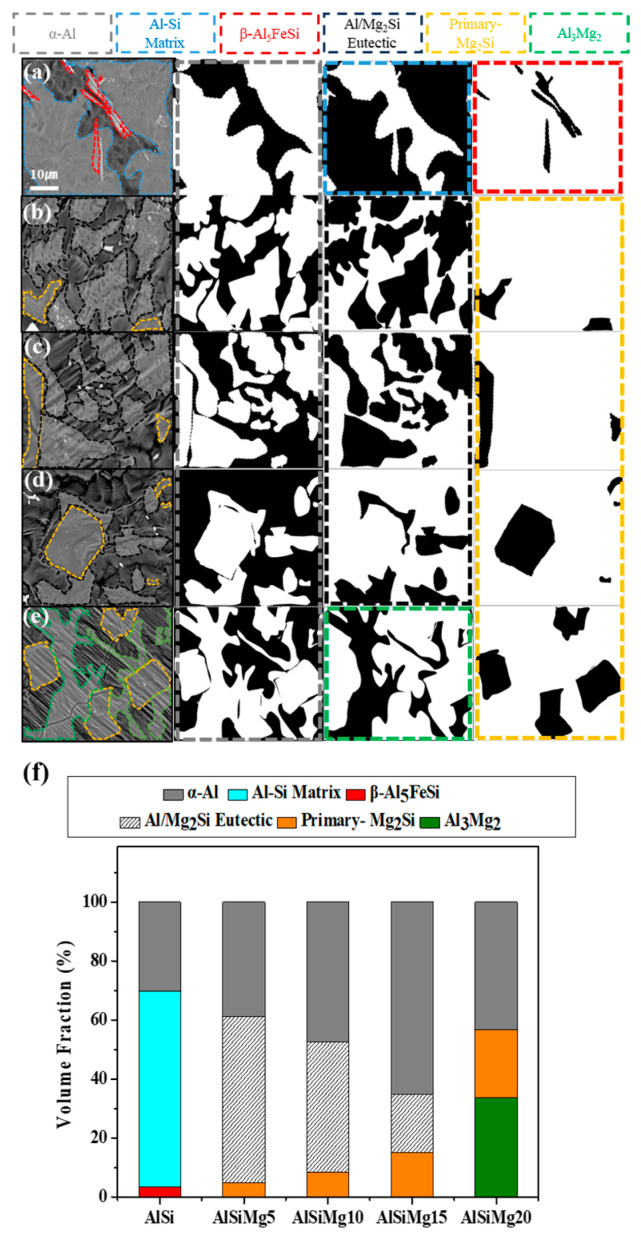
Surface microstructures of the samples with the different phase regions marked: (**a**) AlSi; (**b**) AlSiMg5; (**c**) AlSiMg10; (**d**) AlSiMg15; (**e**) AlSiMg20. (**f**) Volume-fraction plot of the samples.

To analyze the different phases in the coatings, the phases were quantified by estimating their volume fractions using SEM images and image analysis software, as shown in Figure 7 and Table 4. The AlSi sample comprised 66.56% of an Al–Si matrix, along with 30.08% and 3.36% of the α-Al and β-Al_5_FeSi phases, respectively. In AlSiMg5, the Al–Si matrix was transformed into a fine Al/Mg_2_Si eutectic mixture, comprising ~56.35% of the sample. With the increase in the Mg content in the samples, the volume fraction of this Al/Mg_2_Si eutectic mixture gradually decreased, and it completely disappeared in AlSiMg20. Notably, the volume fraction of the primary Mg_2_Si phase increased linearly with increasing Mg content of the Al–Si–xMg alloy; the highest volume fraction (22.99%) was observed in AlSiMg20. Interestingly, an additional Al_3_Mg_2_ alloy phase was observed only in the AlSiMg20 sample (comprising ~33.72% of the sample). As shown in Figure 7f, the AlSiMg5 sample contained the highest volume fraction of the Mg_2_Si-containing phase (from both the primary Mg_2_Si phase and Al/Mg_2_Si eutectic mixture) among the analyzed samples; therefore, it was expected to provide the most sacrificial active sites, significantly delaying the dissolution of Al [44].

### 3.5. Coating Adhesion

**Figure 8:**
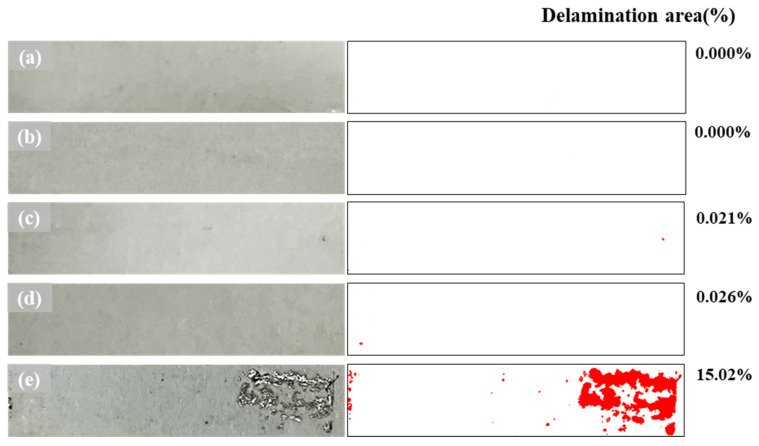
Coating-adhesion evaluation with an attached tape (on the **left**) and the corresponding peeled-off area fractions (on the **right**) for: (**a**) AlSi; (**b**) AlSiMg5; (**c**) AlSiMg10; (**d**) AlSiMg15; (**e**) AlSiMg20.

An adhesive tape was attached to the bent surface of the samples to evaluate their coating adhesion. The appearance of the peeled-off coating layers upon tape removal is shown in Figure 8. An analysis of the delaminated areas (using an image analyzer) indicated no peeling in the AlSi sample and samples with <15 wt.% of Mg. A delaminated area of 15.02% was observed for AlSiMg20, indicating decreased coating adhesion, possibly owing to the formation of a brittle Al_3_Mg_2_ phase within this coating layer.

### 3.6. Corrosion Performance

#### 3.6.1. Potentiodynamic Polarization

**Figure 9:**
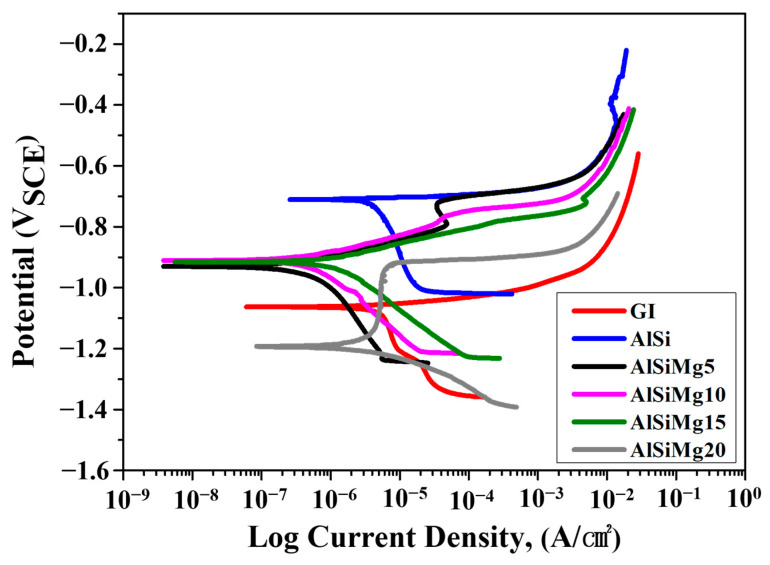
Potentiodynamic polarization plots of hot-dip Al–Si–xMg alloy-coated steel sheets.

The potentiodynamic polarization plots of the Al–Si–xMg samples are shown in Figure 9, and the electrochemical corrosion parameters are listed in Table 5. Hot-dip galvanized (GI) steel sheets were also tested for comparison. A Zn coating layer (approximately 35 μm thick) was produced by immersing the substrate in a pure Zn pot at a temperature of 470 °C for 5 min. The corrosion rate (mpy) was calculated based on the corrosion current density (measured via the Tafel extrapolation method), as follows:

$$\mathrm{Corrosion}\;\mathrm{rate}\;(\mathrm{mpy})=\frac{i_{corr}\times K\cdot EW}{dA}$$

where i_corr_ is the corrosion current density of the sample; K is the corrosion rate constant (1.88 × 10^5^); EW is the equivalent weight; d is the density (g/cm^3^); A is the sample area (cm^2^). The corrosion potential of the Mg-added samples shifted negatively (−0.911 to −1.190 V_SCE_) compared with that of AlSi (−0.711 V_SCE_). This corrosion-potential reduction enhances the sacrificial-protection ability of the Mg-added coatings, thereby improving the corrosion resistance of the coated steel substrates. The corrosion current density of the Mg-containing samples decreased with increasing Mg content (from 5 to 15 wt.%). Notably, all the obtained values were lower than the corrosion current density of the AlSi. The current density of a material is analogous to its corrosion rate; consequently, a decrease in the current density indicates a reduced corrosion rate [7,25]. Among all the Al–Si–xMg alloy coatings, AlSiMg5 showed the lowest corrosion rate (0.198 mpy). Notably, both AlSiMg10 (0.239 mpy) and AlSiMg15 (0.431 mpy) exhibited higher corrosion resistance than AlSi (2.115 mpy). This confirms that the addition of Mg (≤15 wt.%) into AlSi improved its corrosion resistance. The higher corrosion resistance of AlSiMg5, AlSiMg10, and AlSiMg15 than that of AlSi can be attributed to the presence of a fine, uniformly distributed Al/Mg_2_Si eutectic mixture in the coatings, which facilitates the formation of stable corrosion products on the coating-layer surface [44]. In contrast to the other Mg-added samples, AlSiMg20 exhibited a significantly lower corrosion resistance than AlSi. This can be attributed to the corrosion damage incurred by the formation of an Al_3_Mg_2_ phase that is highly susceptible to intergranular corrosion and stress-corrosion cracking [30,31,32,33,34].

#### 3.6.2. EIS Study

**Figure 10:**
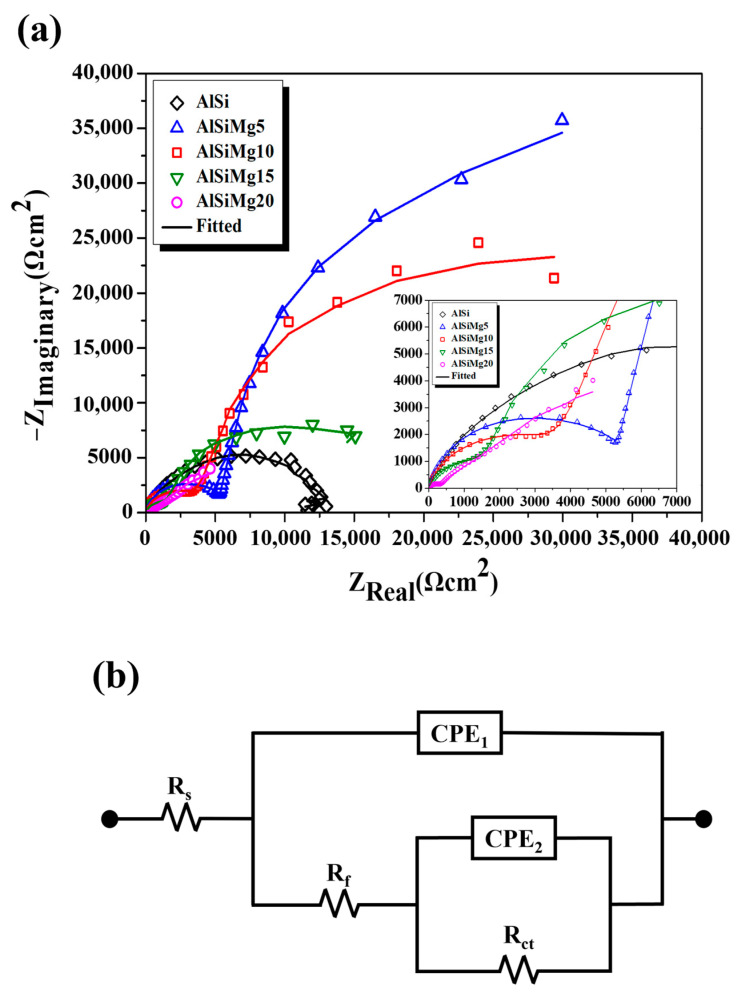
(**a**) Nyquist plots, (**b**) equivalent circuit used to fit EIS results of hot-dip Al–Si–xMg alloy coatings.

Two time constants were observed in the Nyquist plots of each coating (Figure 10a). The first time constant in the high-frequency (HF) region corresponds to the corrosion product formed on the coating-layer surface, whereas the second time constant in the low-frequency (LF) region corresponds to the corrosion resistance at the corrosion product/coating interface [45]. The electrochemical parameters estimated from the fitted EIS results (Figure 10b) of all the samples are summarized in Table 6. The solution resistance (R_s_) of the electrolyte, film resistance (R_f_) of the corrosion product formed, charge transfer resistance (R_ct_) of the double layer, and CPE_1_ and CPE_2_ values, which are constant phase angle elements representing the capacitance of the corrosion product and capacitance of the coating layer, respectively, are listed in Table 6. The CPE can be calculated using equation Z_CPE_ = Y_0_^−1^·(jω)^−n^, where Y_0_ is related to the size of the CPE [Ω^−1^·cm^−2^·s^−n^], j is an imaginary number (j^2^ = −1), and ω is the angular frequency [rad·s^−1^] (ω = 2πf) [46] (n_1_ and n_2_ are constants corresponding to CPE_1_ and CPE_2_, respectively, with values in the range of 0–1). As shown in Figure 10, a large capacitive loop radius was observed in the HF and LF regions of AlSiMg5, indicating the formation of corrosion products with better protective effects than those formed by the other samples. Notably, AlSiMg5 exhibited the highest polarization resistance (R_p_, summation of R_f_ and R_ct_). As the Mg content increased, the diameters of the HF and LF capacitive loops gradually decreased; AlSiMg20 showed the smallest capacitive loop.

#### 3.6.3. Salt Spray Test

**Figure 11:**
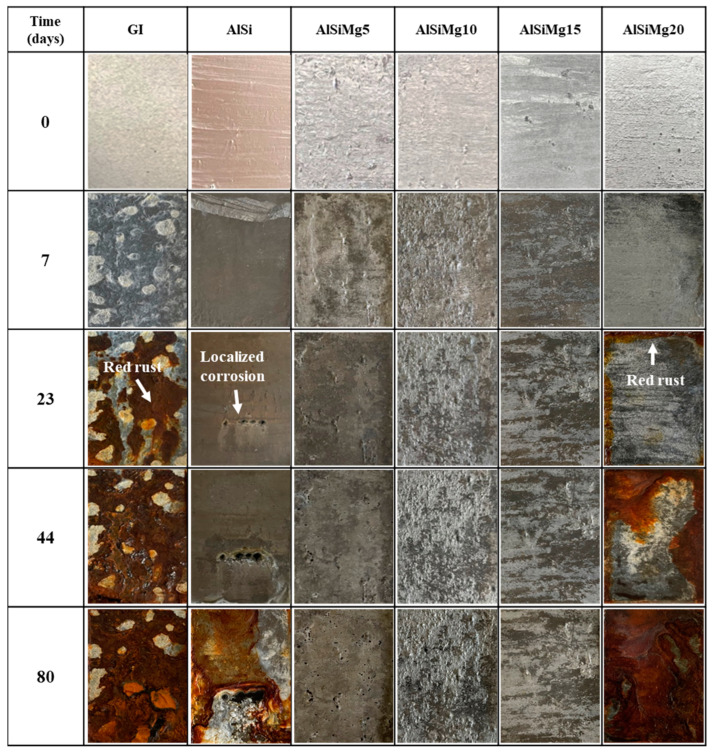
SST results of hot-dip Al–Si–xMg alloy-coated steel sheets.

The long-term corrosion behavior of the Al–Si–xMg coatings was analyzed using 1920 h SSTs; the results are shown in Figure 11. Although the AlSi sample exhibited a better corrosion resistance than the GI sample, localized red-rust formation was observed after 552 h of the SST. This red-rust area rapidly increased with time, and severe red-rust formation was observed after 1920 h. Interestingly, no red-rust formation was observed for AlSiMg5, AlSiMg10, and AlSiMg15, even after 1920 h of the SST. This confirms their excellent corrosion resistance compared to that of AlSi. However, severe red-rust formation occurred in AlSiMg20 after 1056 h, indicating that it has a lower corrosion resistance than AlSi. These results are consistent with the corrosion rates listed in Table 5 and the R_p_ data summarized in Table 6.

## 4. Conclusions

In this study, the effect of the Mg content of hot-dip Al–Si–xMg alloy coatings on their microstructure, electrochemical behavior, and corrosion resistance was investigated. The introduction of Mg into Al–Si coatings increased the surface roughness; the coating layer containing 20 wt.% of Mg showed a significantly higher surface roughness than AlSi. Moreover, the fine eutectic Al/Mg_2_Si phase and coarse primary Mg_2_Si phase coexisted in Al–Si coatings containing ≤15 wt.% of Mg (namely, AlSiMg5 and AlSiMg10). Notably, an additional Al_3_Mg_2_ phase was observed in AlSiMg20. According to the microstructural analysis, among the analyzed samples, AlSiMg5 contained the highest fraction (>60%) of the fine Al/Mg_2_Si eutectic phase, along with a small fraction of the Mg_2_Si single phase. The volume fraction of the primary Mg_2_Si phase increased with increasing Mg content. Electrochemical testing and SSTs were used to assess the corrosion resistance of the coatings. Samples containing 5–15 wt.% of Mg exhibited a higher corrosion resistance than AlSi, with AlSiMg5 exhibiting the most optimized result. Notably, AlSiMg20 showed the lowest corrosion resistance among the analyzed samples (even lower than that of AlSi), which may be attributed to the formation of the Al_3_Mg_2_ phase. We believe that the optimized AlSi-5wt.%Mg alloy-coated steel can be effectively utilized in industries that demand ultra-high corrosion resistance, particularly in energy materials used in marine environments, such as offshore wind power generators and floating solar cell frames.

## Figures and Tables

**Table 1:** Compositions of Al–Si–xMg coatings and their corresponding labels.

Sample	Composition (X-Ray Fluorescence, wt.%)		
	Al	Si	Mg
AlSi	91.11	8.89	-
AlSiMg5	86.51	8.61	4.88
AlSiMg10	82.20	8.25	9.55
AlSiMg15	77.46	7.76	14.78
AlSiMg20	72.99	7.39	19.62

**Table 2:** EDS analysis of the points marked in Figure 3 and their corresponding phases.

Sample	Point	Composition (wt.%)				
		Al	Si	Mg	Fe	Alloy Phase
AlSi	1	52.47	2.41	-	45.12	Fe_2_Al_5_
	2	58.51	9.83	-	31.66	Fe_2_Al_7_Si(τ_5_)
	3	51.13	48.57	-	0.30	Si
	4	96.33	3.40	-	0.27	α-Al
AlSiMg5	1	52.73	1.45	0.02	45.80	Fe_2_Al_5_
	2	64.06	3.83	0.92	31.19	FeAl_3_
	3	86.44	3.85	8.51	1.20	Al/Mg_2_Si Eutectic
AlSiMg10	1	53.27	1.53	0.05	45.15	Fe_2_Al_5_
	2	64.45	1.69	2.05	31.81	FeAl_3_
	3	0.31	62.48	36.59	0.62	Mg_2_Si
	4	85.18	4.99	9.09	0.74	Al/Mg_2_Si Eutectic
AlSiMg15	1	51.28	3.35	-	45.37	Fe_2_Al_5_
	2	62.02	1.43	2.17	34.38	FeAl_3_
	3	0.14	46.36	52.75	0.75	Mg_2_Si
	4	90.27	0.28	8.77	0.68	Al(Mg) Matrix
AlSiMg20	1	52.50	2.37	0.18	44.95	Fe_2_Al_5_
	2	63.35	1.20	2.19	33.26	FeAl_3_
	3	0.80	34.10	64.73	0.37	Mg_2_Si
	4	67.87	0.06	31.86	0.21	Al_3_Mg_2_
	5	86.96	0.42	12.53	0.09	α-Al

**Table 3:** EDS analysis of the points marked in Figure 6, with the corresponding phases.

Sample	Point	Composition (wt.%)				
		Al	Si	Mg	Fe	Alloy Phase
AlSi	1	80.39	18.11	-	1.50	Al/Si eutectic
	2	60.70	17.28	-	22.02	β-Al_5_FeSi
AlSiMg5	1	96.56	1.21	1.22	1.01	α-Al
	2	66.94	13.65	18.52	0.89	Mg_2_Si
AlSiMg10	1	0.80	35.34	63.34	0.52	Mg_2_Si
	2	59.66	16.19	23.43	0.72	Mg_2_Si
	3	91.86	0.57	6.81	0.76	α-Al
AlSiMg15	1	-	36.78	62.70	0.52	Mg_2_Si
	2	88.81	0.01	10.71	0.47	α-Al
AlSiMg20	1	0.91	34.33	63.92	0.84	Mg_2_Si
	2	80.02	0.03	19.24	0.71	α-Al
	3	64.49	0.01	35.15	0.35	Al_3_Mg_2_

**Table 4:** Volume fractions of the different phases in the alloy coating samples.

	Sample	AlSi	AlSiMg5	AlSiMg10	AlSiMg15	AlSiMg20
Phase						
α-Al		30.08	38.74	47.51	62.16	43.28
Al-Si Matrix		66.56	-	-	-	-
β-Al_5_FeSi		3.36	-	-	-	-
Al/Mg_2_Si Eutectic		-	56.35	44.05	19.96	-
Primary-Mg_2_Si		-	4.91	8.44	14.98	22.99
Al_3_Mg_2_		-	-	-	-	33.72

**Table 5:** Electrochemical corrosion parameters of hot-dip Al–Si–xMg alloy-coated steel sheets.

Sample	E_corr_ (V)	i_corr_ (µA/cm^2^)
GI	−1.060	7.690
AlSi	−0.711	4.930
AlSiMg5	−0.930	0.471
AlSiMg10	−0.911	0.558
AlSiMg15	−0.915	0.945
AlSiMg20	−1.190	5.810

**Table 6:** Electrochemical parameters estimated from the fitted EIS results (Figure 10b) of the hot-dip Al–Si–xMg alloy coatings.

Sample	R_s_ (Ω cm^2^)	CPE_1_ (F/cm^2^)	n_1_	R_f_ (Ω cm^2^)	CPE_2_ (F/cm^2^)	n_2_	R_ct_ (Ω cm^2^)	R_p_ (Ω cm^2^)
AlSi	12.75	1.13 × 10^−6^	0.81	3123	8.70 × 10^−6^	1.00	9221	12,344
AlSiMg5	15.56	9.89 × 10^−6^	0.92	5598	3.23 × 10^−4^	1.00	75,580	81,178
AlSiMg10	16.29	3.09 × 10^−5^	0.89	3904	3.21 × 10^−4^	0.98	47,720	51,624
AlSiMg15	14.29	9.60 × 10^−5^	0.86	1686	4.94 × 10^−4^	0.98	15,970	17,656
AlSiMg20	16.00	2.08 × 10^−5^	0.86	349	2.83 × 10^−4^	0.77	1866	2215

## Data Availability

Not applicable.
